# Identification of Pathologic Grading-Related Genes Associated with Kidney Renal Clear Cell Carcinoma

**DOI:** 10.1155/2022/2818777

**Published:** 2022-07-30

**Authors:** Weijian Xiong, Jin Zhong, Ying Li, Xunjia Li, Lili Wu, Ling Zhang

**Affiliations:** Nephrology Department of Chongqing Hospital of Traditional Chinese Medicine, China

## Abstract

**Background:**

Renal epithelium lesions can cause renal cell carcinoma. This kind of tumor is common among all renal cancers with poor prognosis, of which more than 70% belong to kidney renal clear cell carcinoma. As the pathogenesis of KIRC has not been elucidated, it is necessary to be further explored.

**Methods:**

The Genomic Spatial Event database was used to obtain the analysis dataset (GSE126964) based on the GEO database, and The Cancer Genome Atlas was applied for KIRC data collection. edgeR and limma analyses were subsequently conducted to identify differentially expressed genes. Based on the systems biology approach of WGCNA, potential biomarkers and therapeutic targets of this disease were screened after the establishment of a gene coexpression network. GO and KEGG enrichment used cluster Profiler, enrichplot, and ggplot2 in the R software package. Protein-protein interaction network diagrams were plotted for hub gene collection via the STRING platform and Cytoscape software. Hub genes associated with overall survival time of KIRC patients were ultimately identified using the Kaplan-Meier plotter.

**Results:**

There were 1863 DEGs identified in total and ten coexpressed gene modules discovered using a WGCNA method. GO and KEGG analysis findings revealed that the most enrichment pathways included Notch binding, cell migration, cell cycle, cell senescence, apoptosis, focal adhesions, and autophagosomes. Twenty-seven hub genes were identified, among which FLT1, HNRNPU, ATP6V0D2, ATP6V1A, and ATP6V1H were positively correlated with OS rates of KIRC patients (*p* < 0.05).

**Conclusions:**

In conclusion, bioinformatic techniques can be useful tools for predicting the progression of KIRC. DEGs are present in both KIRC and normal kidney tissues, which can be considered the KIRC biomarkers.

## 1. Introduction

Renal tubular epithelial lesions can cause renal cell carcinoma (RCC) [1]. Pathological classification of RCC mainly consists of papillary renal cell carcinoma, kidney renal clear cell carcinoma (KIRC), renal collecting duct carcinoma, chromophobe renal cell carcinoma, and unclassified renal cell carcinoma [2]. KIRC takes up around 70% to 80% of RCC [3, 4]. The incidence in males is twice as high as in females, with the peak age at 60-70 years old [5]. KIRC patients may develop early atypical symptoms of mild fever and fatigue or no symptoms. Hemorrhage, cystic degeneration, necrosis, and calcification in advanced tumor tissues are common, which causes the RCC triad: hematuria, flank pain, and flank masses, greatly affecting the patients' quality of life [6].

KIRC is a common subtype of kidney cancer, and it is hard to manage as there are few identified effective therapeutic targets and molecular drugs for KIRC treatment. Lately, its incidence has been increasing gradually. Over the past decade, RCC-associated therapeutic options have changed from nonspecific immune pathways (cytokines) to targeted therapy against vascular endothelial growth factor (VEGF) then to recent novel immunotherapeutic agents [7]. Typically, KIRC is not sensitive to radiotherapy, chemotherapy, and immunotherapy, and surgery is the current primary option against it [1]. The survival time of 60% of KIRC patients is 1 to 2 years after diagnosis, and there are 30% distant metastasis when being diagnosed [8]. Hence, effective therapeutic targets and prognostic molecular markers are helpful for early diagnosis of KIRC, providing evidence for early intervention treatment.

The Cancer Genome Atlas (TCGA) database is available for data retrieval related to clinical case, gene mutation, mRNA expression, miRNA expression, and methylation. The platform is recognized as an important data source for cancer research. Gene Expression Omnibus (GEO) is also extensively applied as an online cancer research data repository related to high-throughput gene expression data globally. This research screened differentially expressed genes (DEGs) through TCGA and GEO data mining in KIRC and normal kidney tissues, which were analyzed utilizing several bioinformatic analyses including PPI, WGCNA, functional module analysis, and Cytoscape-hub survival analysis, and hub genes related to the regulation of KIRC development were identified ultimately. The findings are expected to identify novel possible prognostic biomarkers and drug target candidate genes for the reference of treating KIRC patients in clinic. This study was aimed at finding new biomarkers related to KIRC tumor growth and clinical parameters to offer evidence for the early diagnosis of KIRC and improve prognosis, thereby providing a theoretical basis for designing new effective diagnosis and treatment programs.

## 2. Methods

### 2.1. Data Collection

The databases TCGA (https://www.cancer.gov/about-nci/organization/ccg/research/structural-genomics/tcga) and GEO (GSE126964) (https://www.ncbi.nlm.nih.gov/geo/query/acc.cgi?acc=GSE126964) were applied for data collection.

### 2.2. Data Analysis

The expression of sample DEGs were analyzed through edgeR and limma packages. *p* values obtained were corrected using multiple hypothesis tests, and the threshold was determined using false discovery rate (FDR). Hence, *q* values indicated the corrected *p* values. The folds of differential expression were also calculated as per fragments per kilobase of exon model per million mapped fragments (FPKM) value, that is, fold change. The overlapped differential results from the edgeR and limma analyses were obtained.

### 2.3. WGCNA Analysis

The WGCNA systematically depicts association patterns of genes in multiple samples through a biological approach and allows to discrimination of highly covarying gene sets. The R software package of WGCNA can realize several functions for module detection, network construction, gene selection, and data simulation, etc. Source codes and software packages can be downloaded at http://www.genetics.ucla.edu/labs/horvath/CoexpressionNetwork/Rpackages/WGCNA [9]. We used the WCCNA R package to construct the coexpression network. First, the samples are clustered to assess the presence of any significant outliers. Second, the coexpression network is constructed using the automatic network construction function. The R function pickSoftThreshold is used to calculate the soft threshold power *β*, and coexpression similarity is proposed to calculate the adjacency.

### 2.4. Module-Trait Relationship

As module eigengene (ME) allows to summarize of modular gene expression, the correlation between ME and input clinical parameters is calculated, which is called module-trait relationship analysis. To clarify the module-trait relationship of gene modules, we first ranked the corresponding module genes as per the constructed modules by WGCNA. ME of each module was calculated and correlated with clinical parameters.

### 2.5. Survival Analysis and DEG Extraction

Survival analysis was conducted employing the software package Survminer. DEGs were analyzed through limma software package, and DEGs were analyzed. DEGs (high score group/low score group) with absolute values of log2 (fold change) more than 1.0 and DEGs with FDR less than 0.05 were considered statistically significant.

### 2.6. GO and KEGG Enrichment Analyses

The obtained DEGs were analyzed through GO and KEGG enrichment using cluster Profiler, enrichplot, and ggplot2 in the R software package. The *p* and *q* values of enriched pathways (<0.05) were considered significantly enriched.

### 2.7. PPI Network Construction and Hub Gene Screening

STRING platform [10] (http://string-db.org) was employed for PPI construction. All DEGs were imported into the STRING database, with the confidence levels more than or equal to 0.4 considered significant. Meanwhile, a PPI network was constructed using the STRING platform and Cytoscape 3.6.1 for protein-molecular interaction visualization. Degrees between the genes were calculated based on degree algorithm using the plugin Cytohubba of Cytoscape; thereby, the hub genes are screened from the PPI network. The Gene Expression Profiling Interactive Analysis (GEPIA) web server (http://gepia.cancer-pku.cn/detail.php?gene=fabp5) [11] was used to analyze the hub DEGs, and the survival curve was plotted subsequently in terms of the association of gene expression and prognosis under a set condition as log2FC < 1 and *p* < 0.05.

## 3. Results

### 3.1. The KIRC DEGs Are Screened from the GEO-TCGA Database

**Figure 1:**
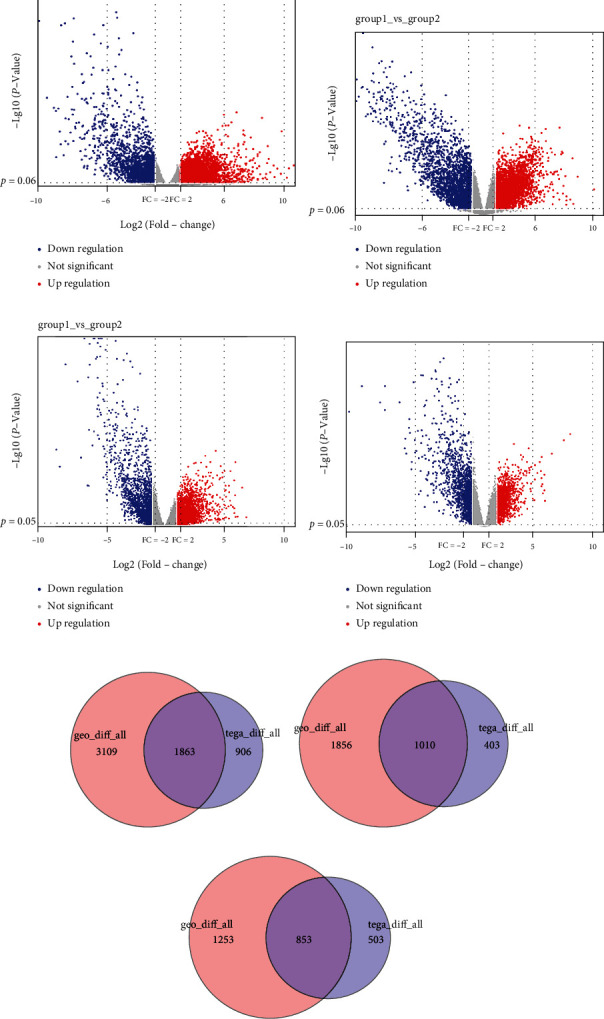
DEGs screening. DEGs of cancer patients and healthy group are screened from GEO database using (a) edgeR and (b) limma. DEGs of cancer patients and normal groups are screened from TCGA database using (c) edgeR and (d) limma. Overlapped DEGs of all (e), upregulated (f), and downregulated (g) are obtained from GEO and TCGA databases.

An expression analysis dataset GSE126964 was exported from GEO database and DEGs of KIRC, and healthy people were obtained and analyzed utilizing edgeR and limma to collect overlapped genes. There were 4972 DEGs screened with 2866 upregulated and 2106 downregulated (Figures 1(a) and 1(b)). Transcriptome data of 528 KIRC tumor samples and 71 adjacent healthy tissues were collected from TCGA database, and DEGs were analyzed using edgeR and limma, and overlapped genes were obtained. There were 2769 DEGs screened with 1413 upregulated and 1356 downregulated (Figures 1(c) and 1(d)). There were a total of 1863 overlapped DEGs obtained from the two databases with 1010 upregulated and 853 downregulated (Figures 1(e)–1(g)).

### 3.2. Construction of WGCNA

**Figure 2:**
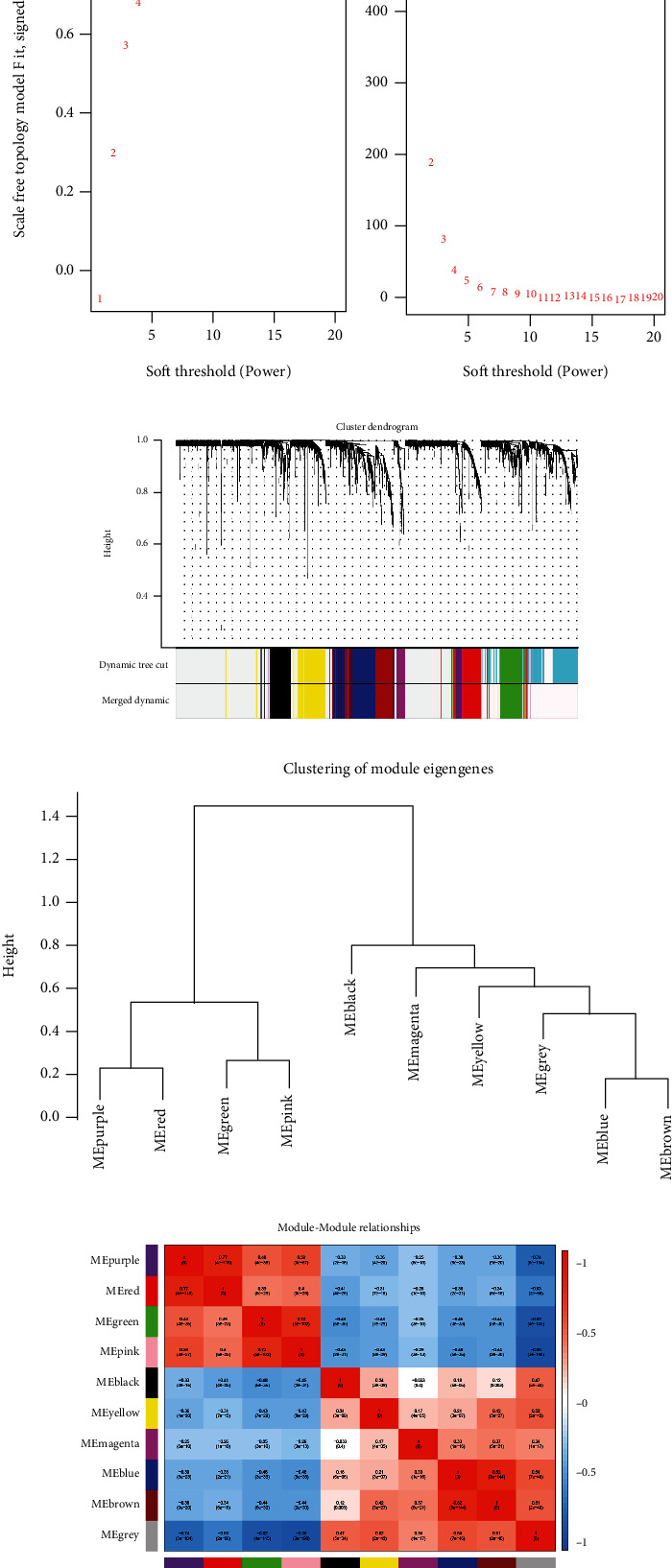
DEGs were analyzed using the WGCNA and gene clustering tree analyses of module eigengenes. (a) Scale-free exponential analysis of various soft threshold powers (*β*). (b) Clustered module dendrogram (top) and colored bands (bottom) of DEGs, and each dendrogram represents module color. (c) Gene cluster dendrogram based on dissimilarity of topological overlap and module color assignment. (d) Correlation analysis of modules. ME: module eigengenes.

Based on the systems biology approach of WGCNA, a diagram of gene coexpression network was constructed, and potential biomarkers and therapeutic targets of this disease were screened. In our study, a weighted gene correlation network was constructed using 1863 DEGs and 528 KIRC samples. The soft threshold power *β* was calculated first, coexpression similarity was elevated to calculate the adjacency, and the WGCNA network was ultimately established. By using WGCNA pickSoftThreshold function, network topology analysis was performed. The soft threshold power *β* was adjusted to 14 in subsequent analysis, and average connectivity was relatively high when scale independence was 0.9 (Figure 2(a)). Gene networks were constructed, and modules were identified using the WGCNA R package. As shown in Figure 2(b), there were ten identified coexpressed gene modules with different colors. Gray defaulted genes that could not be classified into any module. When there are too many genes in the gray module, the previous procedures of expression matrix for gene screening may not be appropriate (Figure 2(b)). MEgray contained genes that did not belong to any module, and it was the largest module. The connectivity of eigengenes was analyzed, which provided pair association of gene coexpression modules. Based on eigengene clustering, the 10 modules could be classified into two groups (Figure 2(c)). Among the modules, MEbrown had a significant positive correlation with MEblue, whereas MEpink was negatively correlated with MEblack, MEblue, and MEbrown (Figure 2(d)).

### 3.3. Module-Trait Relationship Validation

**Figure 3:**
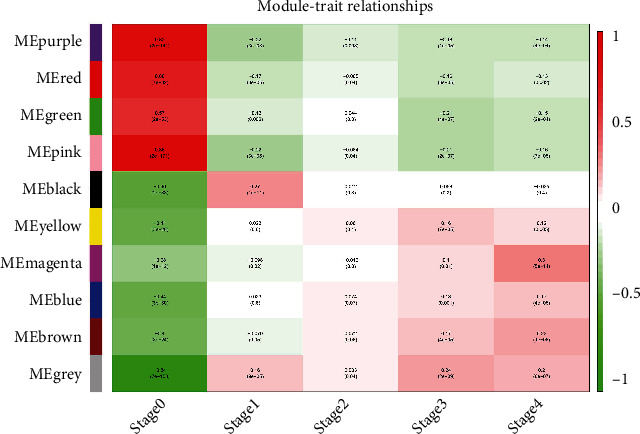
Common module eigengenes and module-clinical signature relationships at different stages of KIRC. The chart rows correspond to different ME, and the columns correspond to clinical parameters. The cell numbers indicate the corresponding correlation and *p* values. Colors of each cell were filled based on correlation and the color legend. The strength and direction of the correlation are shown in the heatmap on the right panel. ME: module eigengenes.

WGCNA can also be used for correlation analysis (*r* values) between modules and clinical parameters. In terms of gene expression profiles by module eigengenes (ME), its correlation with input clinical parameters was analyzed, which is called module-trait relationship analysis. To identify module-trait relationships of gene modules, we assigned genes into corresponding modules with reference to the initially constructed modules. The correlation of each module with clinical parameters was calculated using a function of WGCNA module eigengene [12]. Additionally, the difference is statistically significant if *p* values < 0.05. MEpink has a positive correlation with early KIRC (*r* = 0.85, *p* = 2 × 10^−171^) but has a negative correlation with third-stage KIRC (*r* = −0.21, *p* = 2 × 10^−7^), while MEgreen was also negatively related to KIRC at the third stage (*r* = −0.21, *p* = 4 × 10^−7^) but positively related to KIRC at the first stage (*r* = 0.27, *p* = 1 × 10^−5^); MEmagenta has a positive correlation with the fourth KIRC stage (*r* = 0.3, *p* = 5 × 10^−14^) (Figure 3). As early KIRC is often asymptomatic, it can be discovered only when the tumor volume is big enough. Major clinical manifestations included renal flank pain, hematuria, and flank mass, and the modules of the other four stages were selected for follow-up analysis.

### 3.4. GO and KEGG Enrichment Analyses

**Figure 4:**
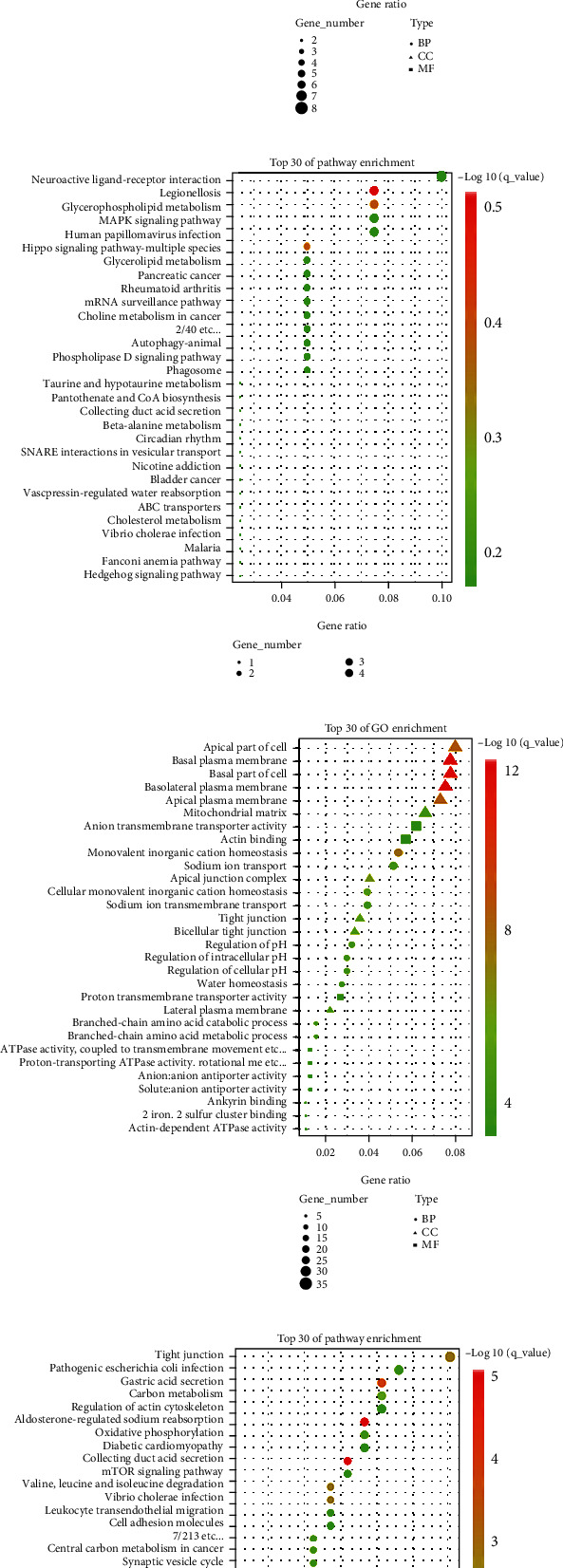
GO and KEGG analyses of module genes. GO terms (a) and KEGG enrichment analysis (b) of MEblack module. GO terms (c) and KEGG enrichment analysis (d) of MEyellow module. GO terms (e) and KEGG enrichment analysis (f) of MEgreen modules. GO terms (g) and KEGG enrichment analysis (h) of MEmagenta module.

To clarify the biological processes (BP), cellular components (CC), and molecular functions (MF) as well as signaling pathways related to module-trait, we applied several modules with significant module-trait relationships for analysis. Most of the MEblack module gene enrichment included ameboidal-type cell migration, extracellular matrix organization, and epithelial cell migration in BP. Collagen-containing extracellular matrix was enriched in CC; extracellular matrix structural constituent was enriched in MF (Figure 4(a)). The mediated signaling pathways included the Notch signaling pathway, Rap1 signaling pathway, PI3K-Akt signaling pathway, and focal adhesion (Figure 4(b)).

MEyellow module genes were mostly enriched in positive regulation of MAP kinase activity and necrotic cell death in BP, protein serine/threonine kinase activity in MF, and nuclear speck, presynapse, and autophagosome in CC (Figure 4(c)). The mediated signaling pathways included glycerophospholipid metabolism and MAPK signaling pathway (Figure 4(d)).

MEgreen module genes were mainly enriched in monovalent inorganic cation homeostasis and sodium ion transport in BP; MF were anion transmembrane transporter activity and actin binding; CC included mitochondrial matrix, basolateral plasma membrane, apical plasma membrane, and basal part of cell (Figure 4(e)). The main mediated signaling pathways were tight junction and mTOR signaling pathway (Figure 4(f)).

MEmagenta module genes were mainly enriched in organelle fission, nuclear division, and chromosome segregation in BP; condensed chromosome, chromosomal region, and spindle in MF; and microtubule binding and tubulin binding in CC (Figure 4(g)). The mediated signaling pathways included cell cycle, p53 signaling pathway, cellular senescence, microRNAs in cancer, and FOXO signaling pathway (Figure 4(h)).

### 3.5. PPI Network Construction and Key Gene Screening

**Figure 5:**
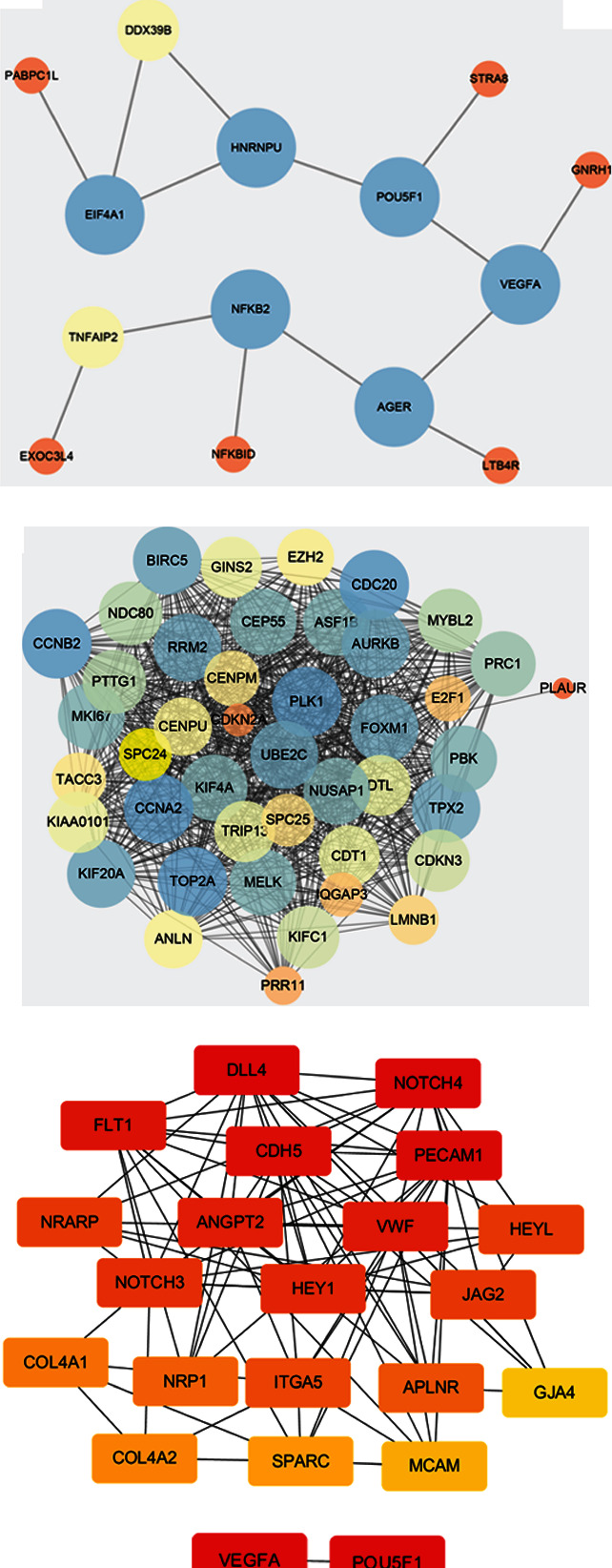
Construction of a PPI network diagram and key gene screening. (a and e) PPI network of DEGs in the MEblack module. (c and f) PPI network of DEGs in the MEyellow module. (b and g) PPI network of DEGs in the MEgreen modules. (d and h) PPI network of DEGs in the MEmagenta module.

To identify key genes, DEGs of several modules with significant module-trait relationships were analyzed using STRING. Meanwhile, a diagram of PPI network was built through Cytoscape. Hub genes of the PPI network diagram were screened out using degree algorithm of a Cytoscape plugin Cytohubba. Five key genes, DLL4, NOTCH4, FLT1, CDH5, and PECAM1, were obtained in the MEblack module (Figures 5(a) and 5(e)). Key genes from the MEyellow module were VEGFA, POU5F1, AGER, NFKB2, EIF4A1, and HNRNPU (Figures 5(c) and 5(f)). Key genes from the MEgreen module included ATP6V0D2, ATP6V1A, ATP6V1H, ATP6V0A4, ATP6V1C2, and ATP6V1B1 (Figures 5(b) and 5(g)). Key genes from the MEmagenta module were KIF20A, UBE2C, CCNA2, TOP2A, PLK1, RRM2, CDC20, AURKB, CCNB2, and TPX2 (Figures 5(d) and 5(h)).

### 3.6. Hub Gene Expression Analysis

**Figure 6:**
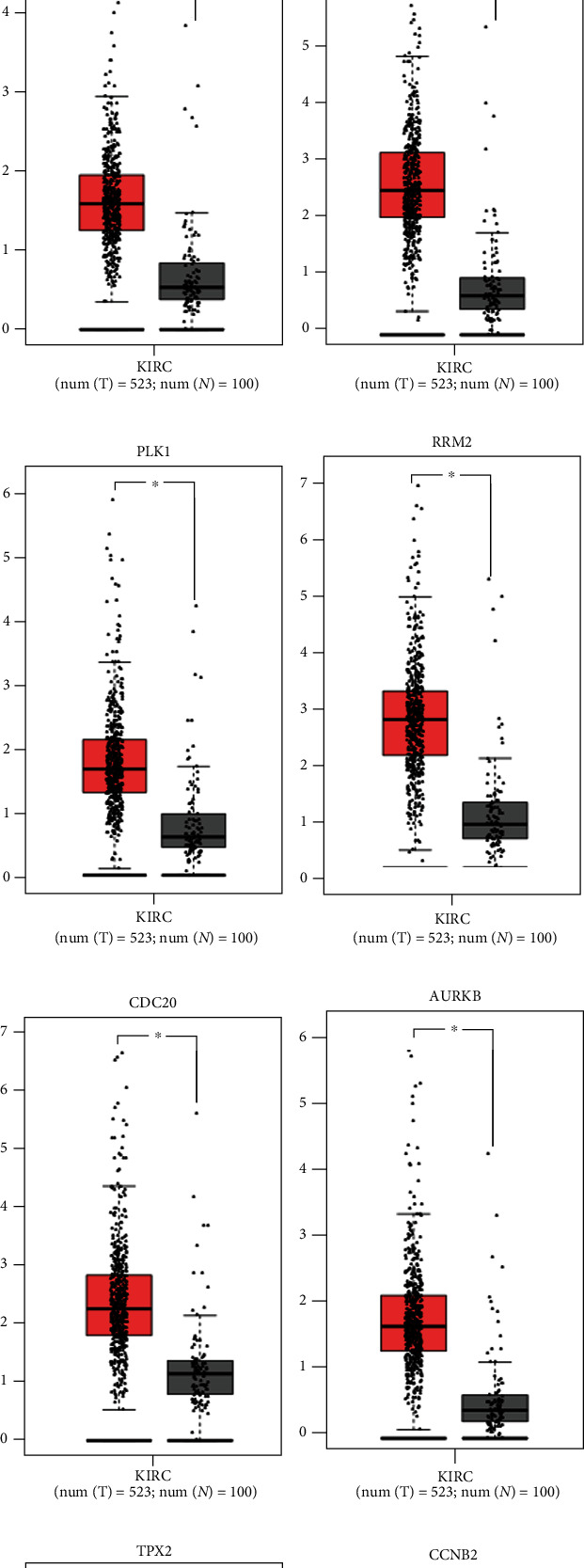
Key gene expression analysis in MEblack, MEyellow, MEgreen, and MEmagenta modules. (a) KIF20A, (b) UBE2C, (c) CCNA2, (d) TOP2A, (e) PLK1, (f) RRM2, (g) CDC20, (h) AURKB, (i) TPX2, and (j) CCNB2. Red: KIRC group; gray: normal group.

The levels of the five hub genes DLL4, NOTCH4, FLT1, CDH5, and PECAM1 in the MEblack module were markedly increased in the KIRC group vs. normal (*p* < 0.05, Figure S1), and those of VEGFA, POU5F1, AGER, and NFKB2 in the MEyellow module were also markedly increased in the KIRC group (*p* < 0.05, Figure S2). The expression of key genes ATP6V0D2, ATP6V1A, ATP6V1H, ATP6V0A4, ATP6V1C2, and ATP6V1B1 in the MEgreen module in the KIRC group was substantially lower vs. normal (*p* < 0.05, Figure S3). The expression of KIF20A, UBE2C, CCNA2, TOP2A, PLK1, RRM2, CDC20, AURKB, CCNB2, and TPX2 in the MEmagenta module in KIRC tissues was markedly higher vs. normal (*p* < 0.05, Figure 6).

### 3.7. Overall Survival (OS)

**Figure 7:**
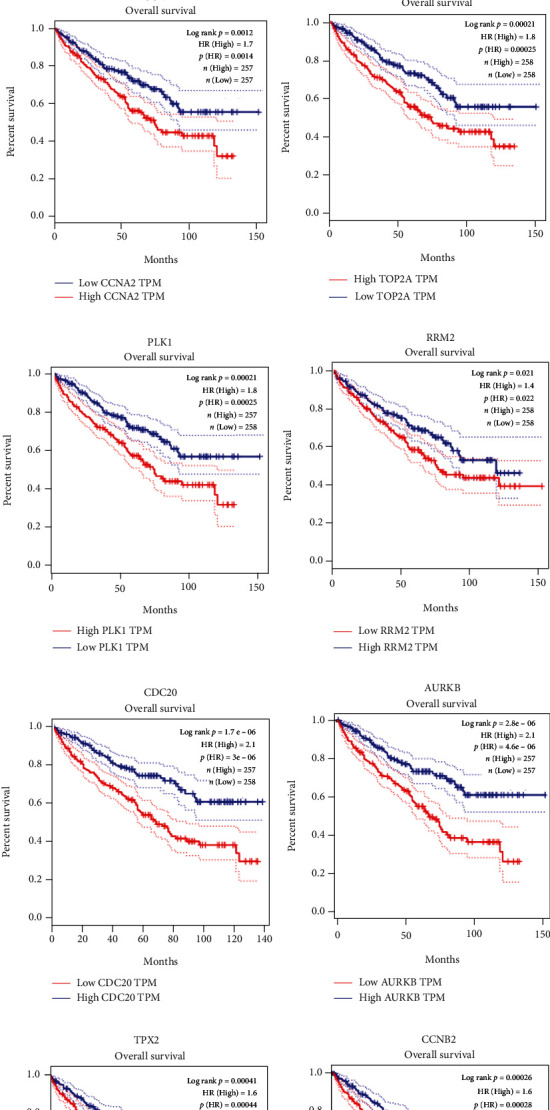
OS analysis of ten hub genes in KIRC (TCGA data in GEPIA). The expression of (a) KIF20A, (b) UBE2C, (c) CCNA2, (d) TOP2A, (e) PLK1, (f) RRM2, (g) CDC20, (h) AURKB, (i) TPX2, and (j) CCNB2 was highly related to OS rates of KIRC patients.

The results of expression survival analysis showed that FLT1, HNRNPU, ATP6V0D2, ATP6V1A, and ATP6V1H were positively correlated with the OS rate of KIRC patients (*p* < 0.05, Figure S4, S5, S6). AGER, ATP6V1C2, KIF20A, UBE2C, CCNA2, TOP2A, PLK1, RRM2, CDC20, AURKB, CCNB2, and TPX2 were negatively correlated with the OS rate of KIRC patients (*p*′ < 0.05, Figures S4, S6, and Figure 7).

## 4. Discussion

RCC is a heterogeneous group of tumors; over 70% belongs to the KIRC category [13] with a high mortality. KIRC is most among all RCC victims, and it is characterized by clear cells pathologically. As a frequent urinary system tumor, a majority of KIRC cases are primary malignancies. The metastasis rate is relatively high at the absent of effective cure [14]. Accumulating evidence indicates that KIRC is a cellular metabolic disease characterized by a low rate of early diagnosis, high metastasis, drug resistance, and disappointed prognosis [15, 16]. Therefore, the exploration of the occurrence and development mechanism is of vital importance for KIRC research in a novel perspective. To improve the early diagnosis rate and prognosis of KIRC patients, great efforts have been made in the research and identification of KIRC biomarkers. Unluckily, no specific biomarker has shown satisfactory results in the clinical application of KIRC diagnosis, classification, and prognosis to date [17, 18]. Hence, elucidating the occurrence and development mechanism of KIRC is urgently needed, and identifying its biomarkers is of great significance for early clinical diagnosis and prognosis for the early screening and treatment of KIRC.

The present study performed a systematic biological approach WGCNA to identify DEGs of KIRC and pathological stage-related gene modules. We identified a total of 1863 DEGs and 10 module-trait relationships, including MEpurple, MEred, MEgreen, MEpink, MEblack, MEmagenta, MEyellow, MEgrey, MEblue, and MEbrown, which were positively associated with pathological grades, implying that ME was important in the occurrence and development of KIRC. GO analysis indicated that the genes in the four modules (MEyellow, MEblack, MEmagenta, and MEgreen) significantly associated with grades were mainly binding to Notch and related to cell migration, cell cycle, cell senescence, apoptosis, focal adhesions, and autophagosomes. In KEGG pathway analysis, we found that several module eigengenes were enriched in the cytokine-cytokine receptor interaction, PI3K-Akt signaling pathway, MAPK signaling pathway, Notch signaling pathway, forkhead box O (FOXO) signaling pathway, p53 signaling pathway, and mTOR signaling pathway. We speculate that these modular genes may mediate the occurrence of KIRC through some signaling pathway.

Some studies have indicated that the PI3K/AKT pathway has a specific therapeutic target for KIRC management, which produces possible values for MTOR and/or related pathway inhibitor drugs against the tumor [19, 20]. The NOTCH pathway is oncogenic in T-cell acute lymphoblastic leukemia and head and neck cancer, which has been reported in such tumors with NOTCH mutations activated [21, 22]. The components of this pathway are also active in RCC [23, 24]. The NOTCH pathway has also been reported overexpressed in a majority of KIRC cases [25]. FOXO belongs to the forehead transcription factor family that plays an essential role in cell fate determination. Meanwhile, it plays a key functional role as a tumor suppressor in various cancers. In different types of cancer, the most important pathway interacting with FOXO is the PI3K/AKT pathway [26]. Therefore, the next focus was to identify the hub genes involved in these signaling pathways.

After identifying KIRC-related module eigengenes using GO and KEGG enrichment analyses, the network diagram of the obtained genes was built based on WGCNA and the correlation of module eigengenes was analyzed. Meanwhile, hub genes were identified using the Cytohubba package. Twenty-seven hub genes including DLL4, NOTCH4, FLT1, and CDH5 were identified, and a total of 25 genes were identified closely related to survival rates of KIRC patients, which might be potential biomarkers for prognosis prediction of KIRC patients. Additionally, potential hub gene transcription was validated using GEO database, in which the expression of ATP6V0D2, ATP6V1A, ATP6V1H, ATP6V0A4, ATP6V1C2, and ATP6V1B1 in KIRC tissues was markedly lower than normal tissues. These findings might provide fundamental evidence for potential biomarker identification and/or anticancer target discovery in future research.

It has been implicated that the Dll4-Notch signaling pathway regulates tumor-initiating cells, i.e., cancer stem cells [27, 28], which has been reported to be characterized by self-renewal, tumor initiation, and differentiation. Furthermore, the cells are essential for tumor growth, tumor resistance, and metastasis [29]. Dll4 blockers can effectively resist tumor activity and be helpful for targeting both Dll4 and VEGF signaling pathways, presenting strong potential for RCC management [30]. Although most Notch4-related studies suggest that Notch4 signaling activation is oncogenic, a Notch4-induced tumor suppressor mechanism has also been identified [31]. Abnormal Notch4 signaling transduction can regulate multiple cellular behaviors to initiate cancer occurrence and progression [32]. Vascular endothelial growth factor A serves as an essential angiogenic cytokine in tumor angiogenesis; it is therefore that it can be a potential for the research of cancer therapy [33]. Previous literature has revealed that RRM2 promotes tumorigenesis and progression of several cancers including lungs [34, 35]. CDC20 is one of the cell cyclins, which is highly expressed in most tissues with malignant tumors. CDC20 acts as an independent prognostic factor in colorectal cancer which might be applied as a potential prognostic biomarker according to Wu et al. [36]. The TOP2A gene encodes DNA topoisomerase, and it mediates DNA replication, chromosome segregation, chromatin condensation, and chromosomal structure preservation during cellular biological processes. Highly expressed TOP2A has been reported to promote breast cancer progression [37, 38]. UBE2C, being a member of the E2 family, has a positive correlation with cancer grades and the quality of outcome in various cancers [39, 40].

In conclusion, we identified pathological grade-related gene modules and hub genes utilizing WGCNA approach. After analysis via GO and KEGG pathway enrichment, module eigengenes of GO terms included endothelial cell development, phagosomal acidification, and Notch binding, and KEGG pathways were associated with metabolism. Mechanistically, these molecular genes might contribute to the progression of KIRC by regulating the previously mentioned pathways. In addition, the twenty-seven hub gene mRNA transcripts were validated in KIRC patients based on the GEO database. It is worth mentioning that the key roles of these hub genes in KIRC were only predicted based on WGCNA theory, and if validated, our findings may provide evidence for the investigation of anticancer targets in KIRC patients. Of course, there are shortcomings in this experiment; the screened hub gene was not experimented to verify whether it plays a role in KIRC.

## Data Availability

The data used to support the findings of this study are included within the article.

## Supplementary Materials

Supplementary 1 Figure S1: key gene expression analysis in MEblack. The expression levels of (A) DLL4, (B) NOTCH4, (C) FLT1, (D) CDH4, and (E) PECAM1. Red: KIRC group; gray: normal group.

Supplementary 2 Figure S2: key gene expression analysis in MEyellow. The expression levels of (A) VEGFA, (B) POU5F1, (C) AGER, (D) NFKB2, (E) EIF4A1, and (F) HNRNPU. Red: KIRC group; gray: normal group.

Supplementary 3 Figure S3: key gene expression analysis in MEgreen. The expression levels of (A) ATP6V0D2, (B) ATP6V1A, (C) ATP6V1H, (D) ATP6V0A4, (E) ATP6V1C2, and (F) ATP6V1B1. Red: KIRC group; gray: normal group.

Supplementary 4 Figure S4: OS analysis of ten key genes in MEblack. (A) DLL4, (B) NOTCH4, (C) FLT1, (D) CDH4, and (E) PECAM1.

Supplementary 5 Figure S5: OS analysis of ten key genes in MEyellow. (A) VEGFA, (B) POU5F1, (C) AGER, (D) NFKB2, (E) EIF4A1, and (F) HNRNPU.

Supplementary 6 Figure S6: OS analysis of ten key genes in MEgreen. (A) ATP6V0D2, (B) ATP6V1A, (C) ATP6V1H, (D) ATP6V0A4, (E) ATP6V1C2, and (F) ATP6V1B1.
