# Supplementary material for: Identification of Pathologic Grading-Related Genes Associated with Kidney Renal Clear Cell Carcinoma
Source: J Immunol Res. 2022 Jul 30;2022:2818777. doi: 10.1155/2022/2818777 (PMC9357261; doi:10.1155/2022/2818777)
Supplement: Supplementary 1 — Figure S1: key gene expression analysis in MEblack. The expression levels of (A) DLL4, (B) NOTCH4, (C) FLT1, (D) CDH4, and (E) PECAM1. Red: KIRC group; gray: normal group. [file 2818777.f1.pdf]

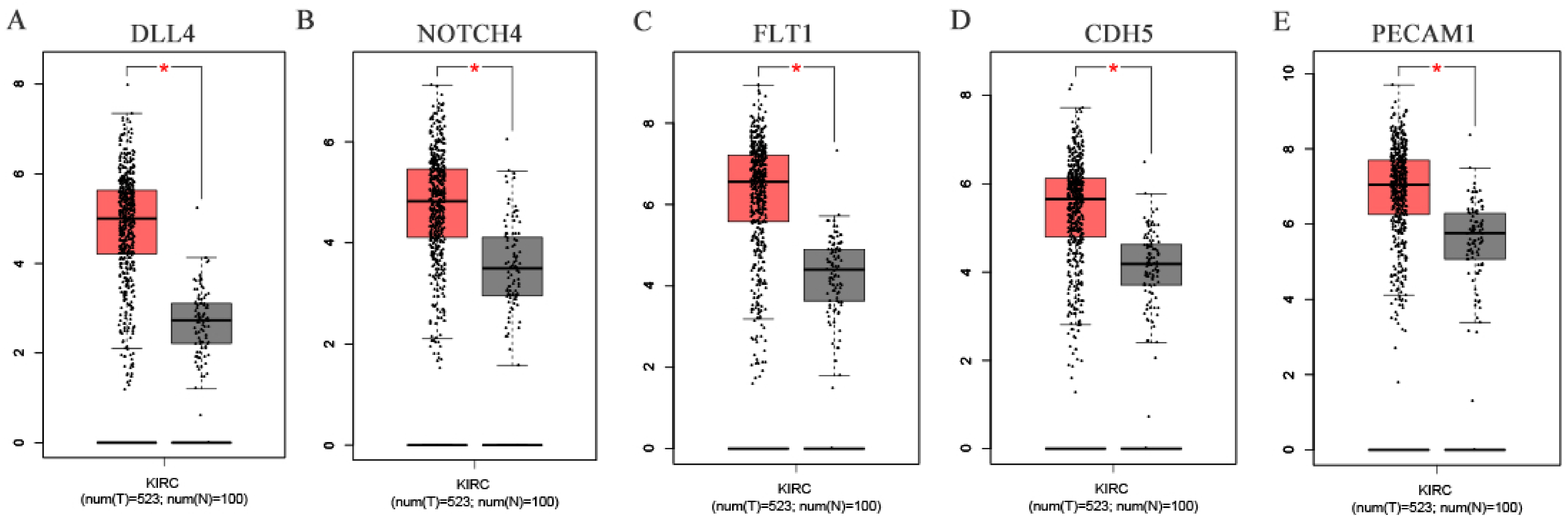

A
DLL4
B
NOTCH4
C
FLT1
D
CDH5
E
PECAM1
KIRC
(num(T)=523; num(N)=100)
