# Supplementary material for: Identification of Pathologic Grading-Related Genes Associated with Kidney Renal Clear Cell Carcinoma
Source: J Immunol Res. 2022 Jul 30;2022:2818777. doi: 10.1155/2022/2818777 (PMC9357261; doi:10.1155/2022/2818777)
Supplement: Supplementary 2 — Figure S2: key gene expression analysis in MEyellow. The expression levels of (A) VEGFA, (B) POU5F1, (C) AGER, (D) NFKB2, (E) EIF4A1, and (F) HNRNPU. Red: KIRC group; gray: normal group. [file 2818777.f2.pdf]

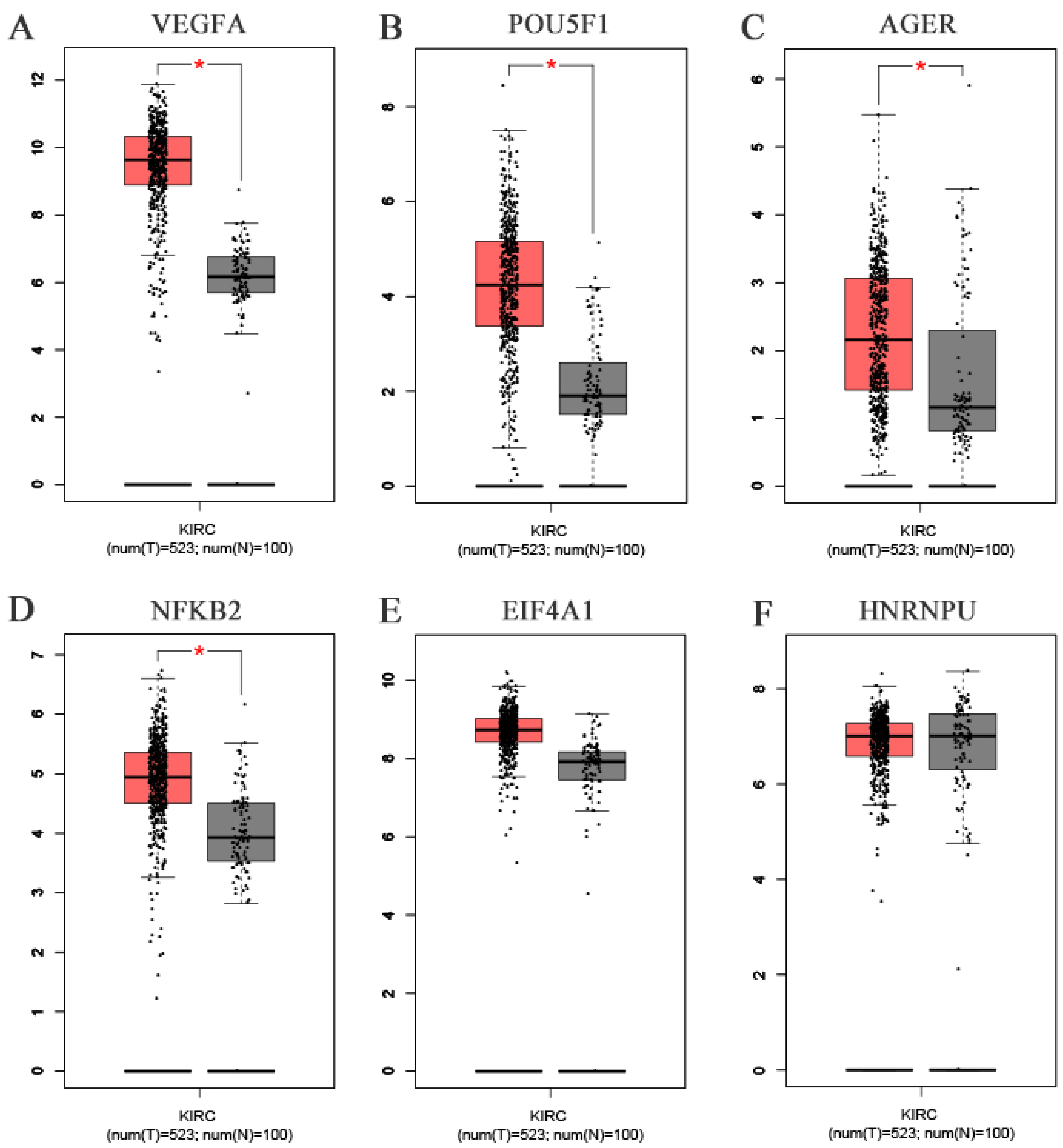

A
VEGFA
B
POU5F1
C
AGER
D
NFKB2
E
EIF4A1
F
HNRNPU
KIRC
(num(T)=523; num(N)=100)
