# Supplementary figures and images for: Identification of Pathologic Grading-Related Genes Associated with Kidney Renal Clear Cell Carcinoma
Source: J Immunol Res. 2022 Jul 30;2022:2818777. doi: 10.1155/2022/2818777 (PMC9357261; doi:10.1155/2022/2818777)

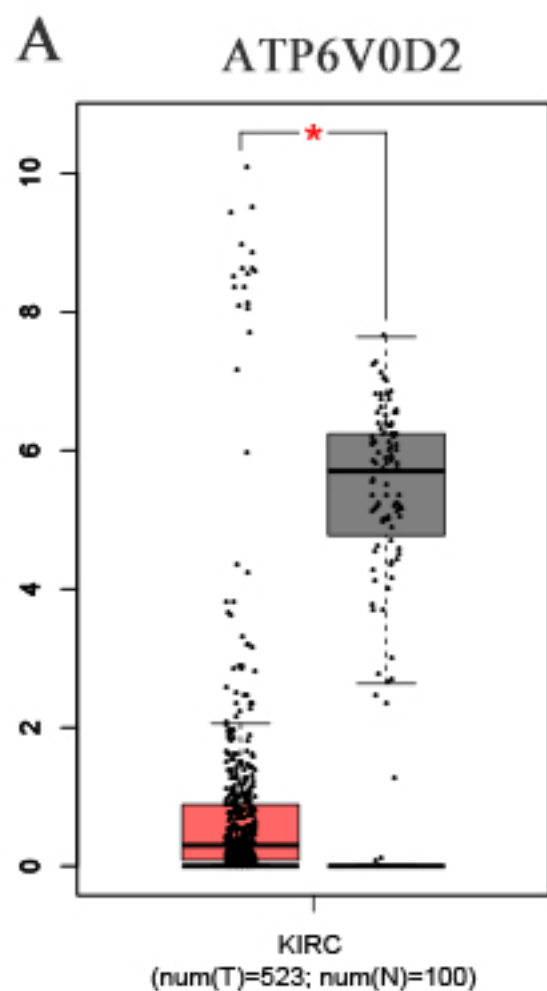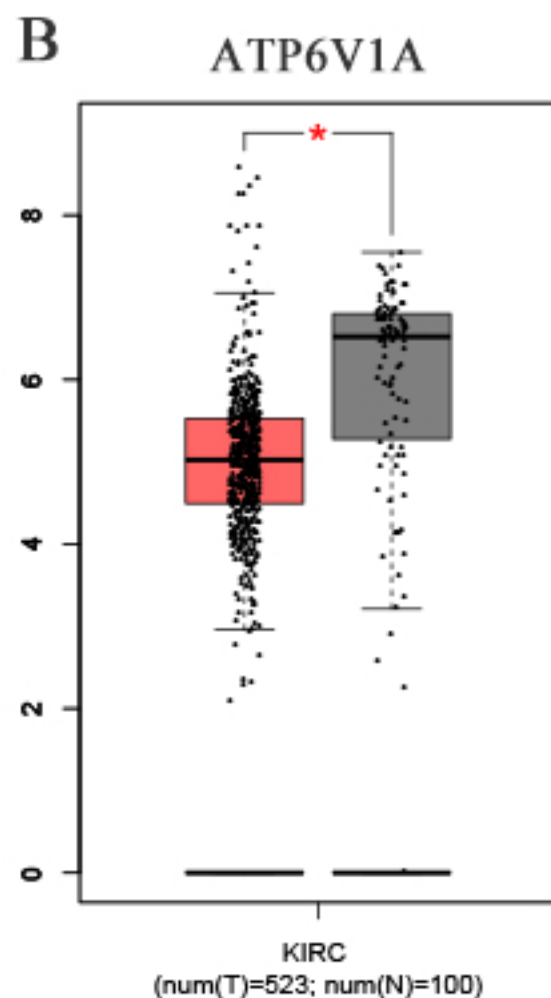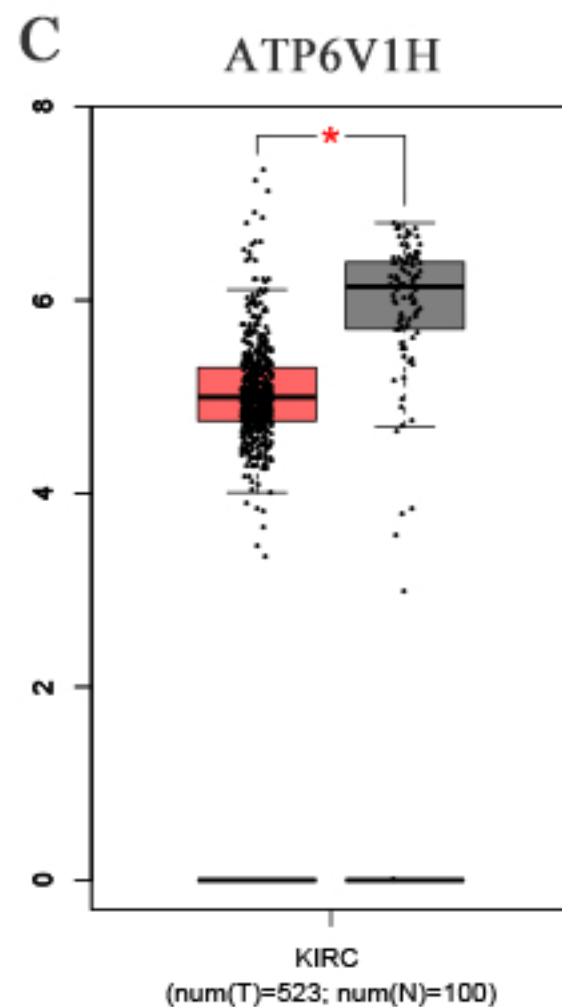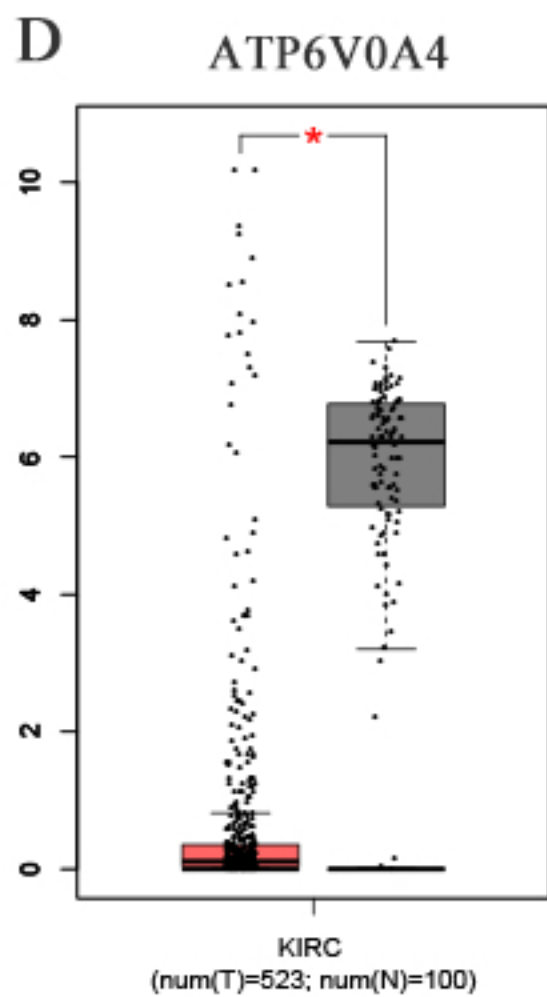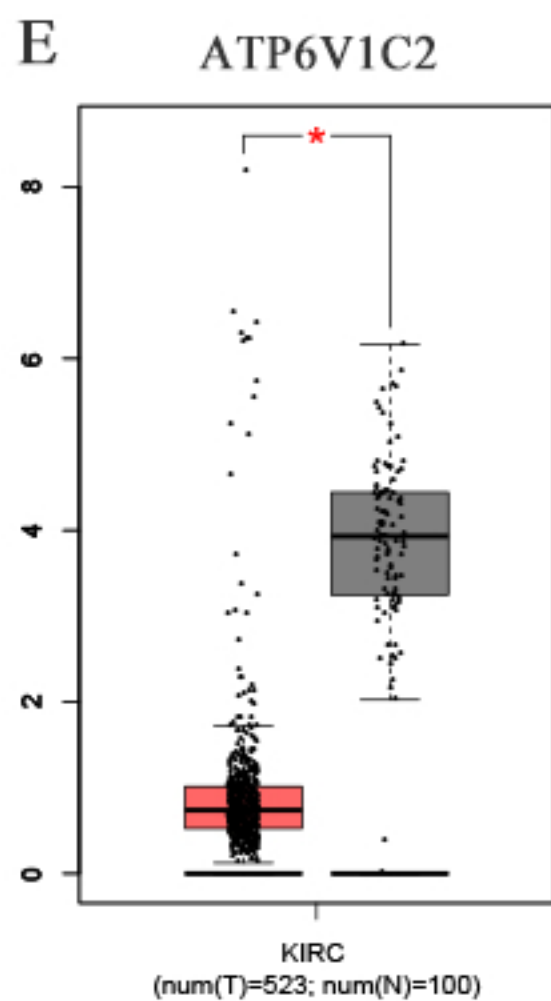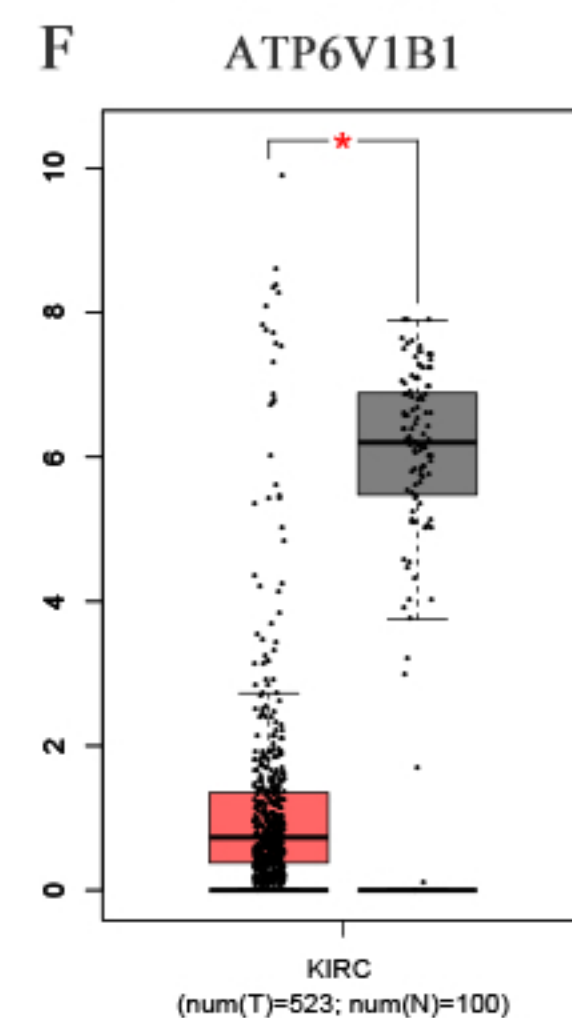

Supplement: Supplementary 3 — Figure S3: key gene expression analysis in MEgreen. The expression levels of (A) ATP6V0D2, (B) ATP6V1A, (C) ATP6V1H, (D) ATP6V0A4, (E) ATP6V1C2, and (F) ATP6V1B1. Red: KIRC group; gray: normal group. [file 2818777.f3.pdf]

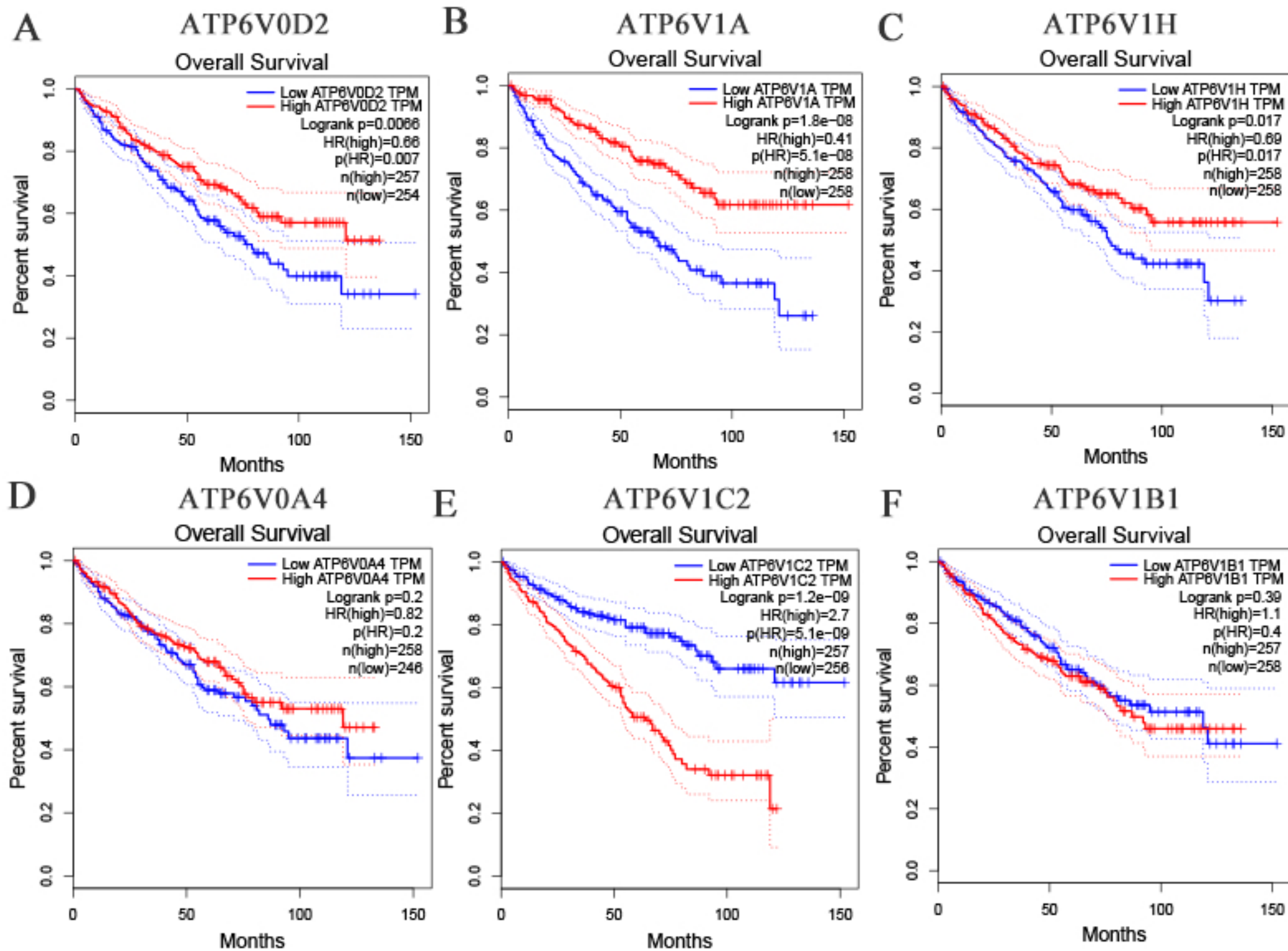

Supplement: Supplementary 6 — Figure S6: OS analysis of ten key genes in MEgreen. (A) ATP6V0D2, (B) ATP6V1A, (C) ATP6V1H, (D) ATP6V0A4, (E) ATP6V1C2, and (F) ATP6V1B1. [file 2818777.f6.pdf]
