# Supplementary material for: Identification of Pathologic Grading-Related Genes Associated with Kidney Renal Clear Cell Carcinoma
Source: J Immunol Res. 2022 Jul 30;2022:2818777. doi: 10.1155/2022/2818777 (PMC9357261; doi:10.1155/2022/2818777)
Supplement: Supplementary 4 — Figure S4: OS analysis of ten key genes in MEblack. (A) DLL4, (B) NOTCH4, (C) FLT1, (D) CDH4, and (E) PECAM1. [file 2818777.f4.pdf]

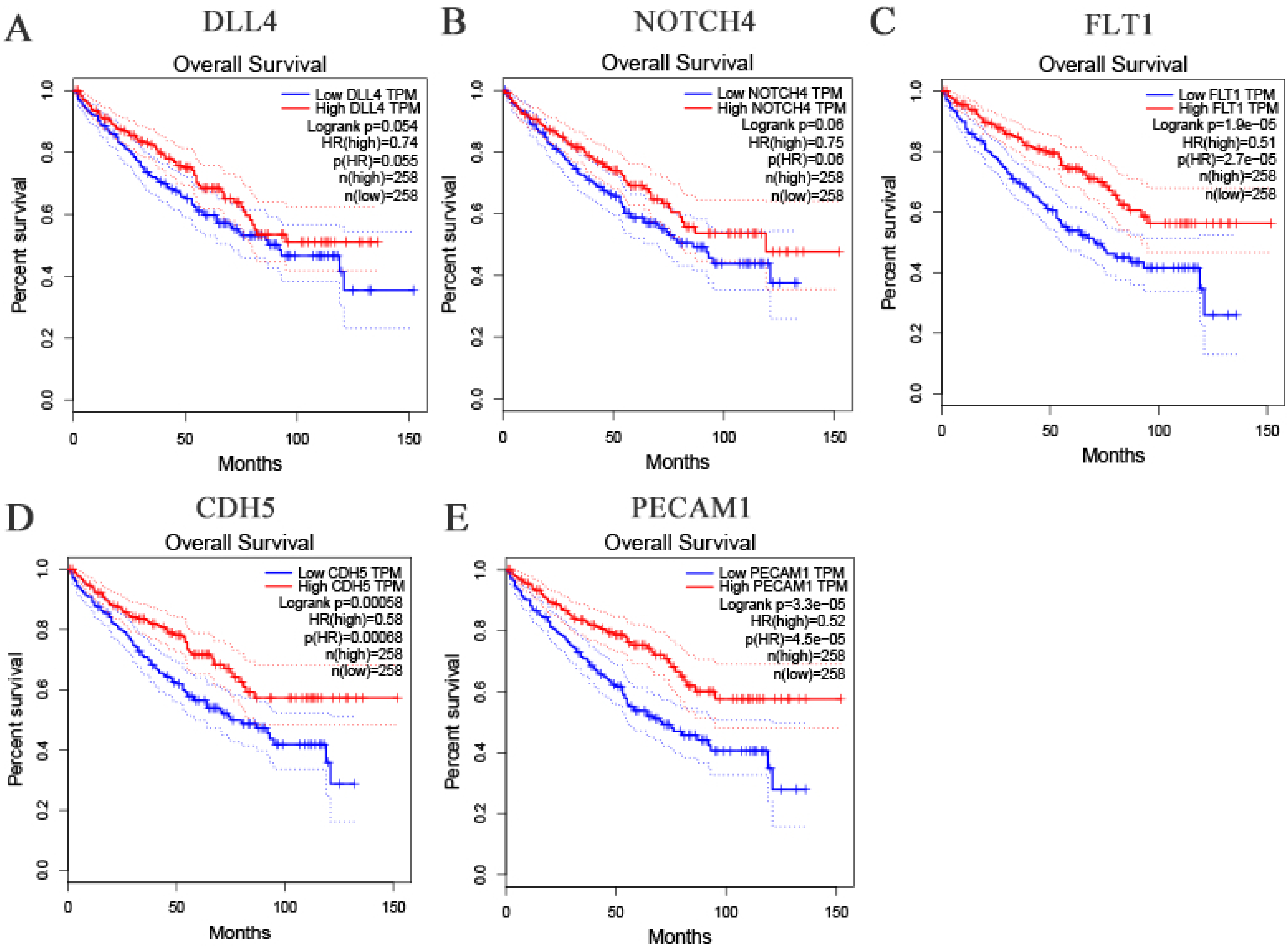
A
DLL4
Overall Survival
Low DLL4 TPM
High DLL4 TPM
Logrank p=0.054
HR(high)=0.74
p(HR)=0.055
n(high)=258
n(low)=258
Percent survival
Months
B
NOTCH4
Overall Survival
Low NOTCH4 TPM
High NOTCH4 TPM
Logrank p=0.06
HR(high)=0.75
p(HR)=0.06
n(high)=258
n(low)=258
Percent survival
Months
C
FLT1
Overall Survival
Low FLT1 TPM
High FLT1 TPM
Logrank p=1.9e−05
HR(high)=0.51
p(HR)=2.7e−05
n(high)=258
n(low)=258
Percent survival
Months
D
CDH5
Overall Survival
Low CDH5 TPM
High CDH5 TPM
Logrank p=0.00058
HR(high)=0.58
p(HR)=0.00068
n(high)=258
n(low)=258
Percent survival
Months
E
PECAM1
Overall Survival
Low PECAM1 TPM
High PECAM1 TPM
Logrank p=3.3e−05
HR(high)=0.52
p(HR)=4.5e−05
n(high)=258
n(low)=258
Percent survival
Months
