# Supplementary material for: Identification of Pathologic Grading-Related Genes Associated with Kidney Renal Clear Cell Carcinoma
Source: J Immunol Res. 2022 Jul 30;2022:2818777. doi: 10.1155/2022/2818777 (PMC9357261; doi:10.1155/2022/2818777)
Supplement: Supplementary 5 — Figure S5: OS analysis of ten key genes in MEyellow. (A) VEGFA, (B) POU5F1, (C) AGER, (D) NFKB2, (E) EIF4A1, and (F) HNRNPU. [file 2818777.f5.pdf]

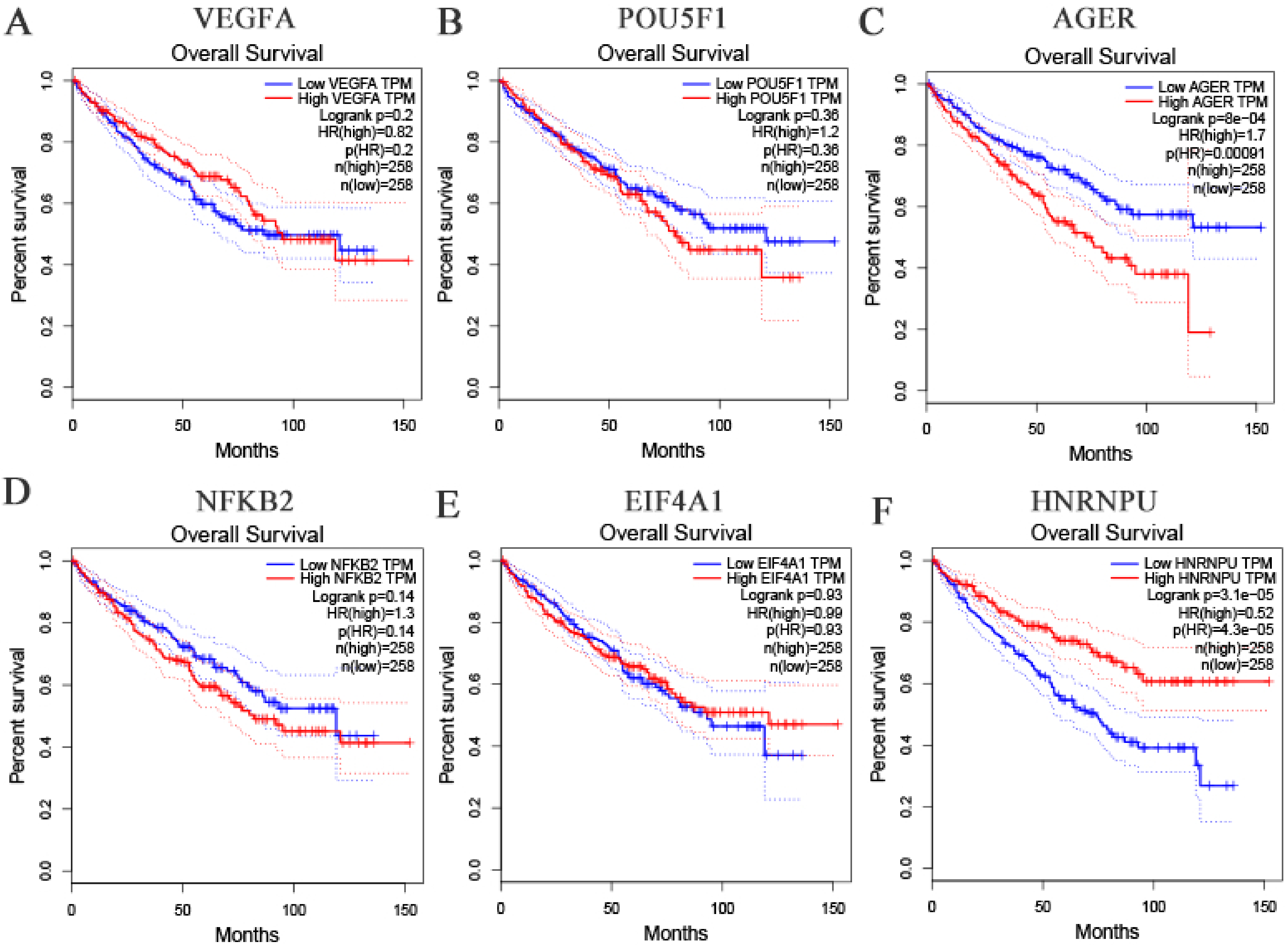
A
VEGFA
Overall Survival
Low VEGFA TPM
High VEGFA TPM
Logrank p=0.2
HR(high)=0.82
p(HR)=0.2
n(high)=258
n(low)=258
Percent survival
Months
B
POU5F1
Overall Survival
Low POU5F1 TPM
High POU5F1 TPM
Logrank p=0.36
HR(high)=1.2
p(HR)=0.36
n(high)=258
n(low)=258
Percent survival
Months
C
AGER
Overall Survival
Low AGER TPM
High AGER TPM
Logrank p=8e−04
HR(high)=1.7
p(HR)=0.00091
n(high)=258
n(low)=258
Percent survival
Months
D
NFKB2
Overall Survival
Low NFKB2 TPM
High NFKB2 TPM
Logrank p=0.14
HR(high)=1.3
p(HR)=0.14
n(high)=258
n(low)=258
Percent survival
Months
E
EIF4A1
Overall Survival
Low EIF4A1 TPM
High EIF4A1 TPM
Logrank p=0.93
HR(high)=0.99
p(HR)=0.93
n(high)=258
n(low)=258
Percent survival
Months
F
HNRNPU
Overall Survival
Low HNRNPU TPM
High HNRNPU TPM
Logrank p=3.1e−05
HR(high)=0.52
p(HR)=4.3e−05
n(high)=258
n(low)=258
Percent survival
Months
